# Investments—What Not to Do

**Published:** 1911-02-15

**Authors:** Henry Hall

**Affiliations:** New York City


### INVESTMENTS—WHAT NOT TO DO.

BY HENRY HALL, NEW YORK CITY.

Dr. Clapp has done me the honor to ask for a few articles on the fundamentals of investments, written for the information of men, usually intensely occupied with the practical work and studies of their professions, and unable, on that account, to devote time and thought to proper consideration of the elaborate array of factors which govern the value and safety of securities.

When a man, whatever his vocation in life, acquires any amount of surplus cash, he is confronted with the question as to whether he shall save it or spend it. People who have large capital can afford to spend. They have passed through the preliminary grind of saving money. They no longer need to be economical. But those who have their fortunes to make, who have not yet provided for old age, must save. The corner stone of ultimate financial comfort is the saving habit. Not in a thousand cases is there more than one exception to this rule. Saving is the starting point in the race for fortune.

It is characteristic of most women, and of too many men, that money burns in their pockets. Even if they do not spend it, they want to put it to immediate use. They want a better return in the way of income than the savings bank supplies, which is perfectly proper. Knowing the fortunes which have been and can be made, in stocks and bonds, they also want to invest their money, at once, in something which will advance in value and thus add to their original capital. This is also perfectly proper and at certain times entirely feasible. Investment at the right time is, in fact, the only way by which the original capital can be increased.

And this leads to the first matter, which should be mentioned with regard to the fundamentals of investment, namely, what not to do.

And first, the investor should begin by disregarding entirely the prospectuses and advertisements of new corporations, no matter how enthusiastic the language of the promoters, and no matter what species of property the corporations have been formed to exploit. The mails and newspapers are full of these alluring invitations to investors. It is natural that a man should want to make his money go as far as possible in his investments. The temptation is almost irresistible to put it into low-priced shares, selling from $1 to $10 each, when a prospectus promises both large future dividends and an advance in price of the shares to double or treble their current value. Owing to this trait of human nature, the people of the United States have been robbed of more than $100,000,000 within the last ten years through putting their money into wildcat propositions, enthusiastically boomed in glowing prospectuses and advertisements. I admit that there are certain low-priced stocks to-day, sound, of genuine merit, selling between $4 and $8 a share, which yield an income of from 10 to 20 per cent. on the investment, and which will ultimately sell much higher than now. But these are not the stocks which are exploited in advertisements.

An investor may take it as an indisputable fact, that no flowery prospectus, no newspaper advertisement and no public promise of any kind is required for the flotation of a valuable business proposition. It is never necessary to call a town meeting, or blow a trumpet, to obtain all the capital that is wanted for a really good thing.

Men of means are ready and eager, always, to supply all the funds required to establish a new and promising business. Take the Mergenthaler Linotype concern as an instance, a company which now pays between 15 and 20 per cent. a year, whose shares it is almost impossible to buy in the market at any time, and never for less than $200 to $220 a share. No advertisement of the stock of that company was ever seen in the newspaper or prospectus. Every share was eagerly taken, at the start, in almost a secret conclave, by men who knew the merits of the machine-This case is typical. Good things do not have to go begging for subscriptions to the capital stock. Flaring advertisements may ordinarily be taken as a sign that important men, sound judges, do not want the stock, and that the promoters of the enterprise have little more in view than to get possession of your money, regardless of what happens afterwards. Thousands of corporations floated in this way have never thereafter been heard of, except discreditably, or except on account of the great decline in the value of their shares. Take the stocks exploited by the ingenious and brilliant Thomas W. Lawson of Boston in his various amazing advertising campaigns! Look at them now! I know of only one, Chino Copper, which has ever made good, or is worth to-day anything like the price which was promised for it. Some of them are worth only a few cents on the dollar.

It has fallen to my lot, at various times within the past year, to be consulted with regard to United Wireless stock. No one ever saw any more wonderful statements as to the prospects of a new company than those which appeared in the circulars with which United Wireless flooded the United States. I do not myself believe that the promoters meant to be dishonest, or that they did not actually hope to establish their company upon a profitable basis, eventually. But if their enterprise had commended itself to the sound judgment of men of capital, they could have placed every dollar of the stock without resorting to glowing advertisements. The United States Government has chosen to regard the company as a swindle and the promoters were recently arrested. A great deal of the public's money has been lost, or indefinitely locked up, in this stock.

In the course of a long experience, nothing has amazed me more than the number of men and women who have allowed themselves to throw away their hard earned money by buying the stock of wild-cat compamies, in reply to prospectuses and advertisements. The stocks are now utterly worthless.

Without dwelling upon this point, in detail, any further, I consider it the first fundamental of investment to pay no attention whatever to securities which can be floated only through the medium of flaming circulars and advertisements.

Next, consult an investment banker, if you wish, but do not take his advice when you buy, until at any rate after you have seen some other people and given the matter a little thought. As a class, the investment bankers are men of the highest sagacity, probity and honor. But they seldom recognize the fact, which is of the utmost importance to a man who wants to make his money grow. They do not admit, to a customer, that s curities can go down, as well as up. Their whole attention is concentrated upon the single question, as to whether the income is safe. Upon that point, they must be considered competent authorities. No one of them will ever imperil his great reputation by intentionally assisting in the flotation of a security, concerning which there is any doubt as to the certainty of the income. But one and all of them will say to a customer, "What difference does the price make to you, as long as the security is for investment?" Well, it does make considerable difference. In order that invested capital may be safe, a bond or a stock must eventually advance in value. To say nothing of profits on the purchase, the price must advance in time to afford a margin of safety for the capital.

It is true that unforeseen calamity may overtake the country, for which no banker, no adviser, and no investor is responsible, and which the best judges could not have foreseen. A man must take his chances as to that. Some things can be foreseen, others not. The San Francisco earthquake in April, 1906, and the sensational failure of the Barings in London in November, 1890, and the unnecessary and intense hostility of the administration at Washington toward legitimate corporations in the early part of 1910, took the whole financial world by surprise, and were each responsible for a serious decline in the prices of securities. No man could have foreseen any one of these things. At any rate, the financial world did not. In this category, I do not rank the great panics which recur at regular intervals, the approach of every one of which is perfectly patent to every student of finance. I mean the accidents, the unforeseen calamities, which occur from time to time, to put to confusion the wisest men.

The point I am trying to make is, that the investment bankers pay no attention to anything except certainty of income; and without the slightest intention to deceive any one they have been responsible many times for loading the investment public with enormous amounts of securities, which afterward declined seriously in price.

A notable instance was the sale of over $300,000,000 worth of 3½ per cent. bonds in 1898-1900 at from $1,020 to $1,120 for each bond. The income thereon was, and always has been, perfectly safe; but those bonds came down after 1901 in a long, sweeping curve, like a skyrocket, until they averaged $825 in 1907, and even now are selling to average $865. The man who bought them above par will never get his money back, or at any rate, not until some distant day, when interest rates on money have fallen permanently to 3 per cent.

So that the time to buy securities is fully as important as safety of income: and an investor should avoid haste and not go into securities at once, simply because he has money available for that purpose.

The affirmative side of what should govern investment will be discussed in a later article.—*Dental Digest.*

